# Victoria continental microplate dynamics controlled by the lithospheric strength distribution of the East African Rift

**DOI:** 10.1038/s41467-020-16176-x

**Published:** 2020-06-08

**Authors:** Anne Glerum, Sascha Brune, D. Sarah Stamps, Manfred R. Strecker

**Affiliations:** 1 Helmholtz Centre Potsdam - GFZ German Research Centre for Geosciences, Potsdam, Germany; 2 University of Potsdam, Potsdam-Golm, Germany; 3 Virginia Tech, Blacksburg, VA USA

**Keywords:** Geodynamics, Structural geology, Geophysics, Tectonics

## Abstract

The Victoria microplate between the Eastern and Western Branches of the East African Rift System is one of the largest continental microplates on Earth. In striking contrast to its neighboring plates, Victoria rotates counterclockwise with respect to Nubia. The underlying cause of this distinctive rotation has remained elusive so far. Using 3D numerical models, we investigate the role of pre-existing lithospheric heterogeneities in continental microplate rotation. We find that Victoria’s rotation is primarily controlled by the distribution of rheologically stronger zones that transmit the drag of the major plates to the microplate and of the mechanically weaker mobile belts surrounding Victoria that facilitate rotation. Our models reproduce Victoria’s GPS-derived counterclockwise rotation as well as key complexities of the regional tectonic stress field. These results reconcile competing ideas on the opening of the rift system by highlighting differences in orientation of the far-field divergence, local extension, and the minimum horizontal stress.

## Abstract (web summary)

One of the largest continental microplates on Earth is situated in the center of the East African Rift System, and oddly, the Victoria microplate rotates counterclockwise with respect to the neighboring African tectonic plate. Here, the authors' modelling results suggest that Victoria microplate rotation is caused by edge-driven lithospheric processes related to the specific geometry of rheologically weak and strong regions.

## Introduction

**Fig. 1:**
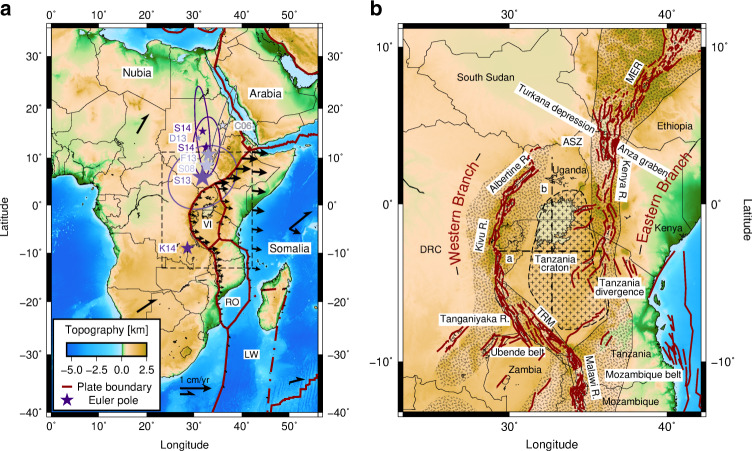
Tectonic setting of the East African Rift System. **a** Plate kinematic configuration of the East African Rift System. Thick red lines represent block model plate boundaries^2^, including the Victoria (VI), Rovuma (RO), and Lwandle (LW) microplates. Black arrows indicate the absolute^7^ (halved vector heads) and relative^3^ (full vector heads) plate motions, albeit depicted in a different scale. Relative velocity vectors along the plate boundaries represent motion with respect to the plate to the west of the boundary; other relative motions are with respect to Nubia. The dashed black box indicates this study's model domain as well as the area shown in panel **b**. Purple stars indicate Euler poles describing the rotation of VI with respect to Nubia: C06^1^, S08^2^, S13^5^, D13^4^, F13^6^, S14^3^, and K14^7^. **b** Zoom-in showing individual rift faults from the GEM fault database^8^, forming the Western and the Eastern Branch. The Tanzania craton (crosses) is outlined in black dashes^9^; other dashed areas indicate the Proterozoic mobile belts^10^. The background in both panels shows the bedrock topography^11^. ASZ Aswa Shear Zone, TRM Tanganyika–Rukwa–Malawi rift segment, MER Main Ethiopian Rift.

The East African Rift System (EARS) is the largest Cenozoic continental rift system. Its Eastern Branch stretches from Afar to the Tanzania divergence, while its western branch stretches from northern Uganda to Mozambique. These two rift branches accommodate divergence between the major Nubian and Somalian plates, and together with diffuse zones of deformation in the southwest Indian Ocean, they delineate the Victoria (hereafter referred to as Victoria), Rovuma, and Lwandle microplates^1,2^ (Fig. 1a). Global positioning system (GPS) and earthquake slip data allowed for quantifying the current motion of all involved plates^1–3^, demonstrating that Somalia, Rovuma, and Lwandle rotate clockwise with respect to Nubia. It, however, also became clear that Victoria’s rotation constitutes a remarkable exception^1,3–6^. The Victoria microplate was found to rotate counterclockwise with respect to the Nubian plate around an Euler pole several hundred kilometers north of the plate (see black arrows and purple stars in Fig. 1a).

The driving mechanism behind the rotation of the Victoria microplate, outlined by faulting, seismicity, and volcanism, has remained enigmatic^3^. It has been suggested that northeastward asthenospheric flow could exert basal drag on the keel of the Tanzania craton^1^, which underlies large parts of the Victoria microplate (Fig. 1b). Interpreting numerical models of the EARS, other studies inferred that asymmetrical plume or double-plume impingement on the keel of the Tanzania craton might generate sufficient torque to drive Victoria rotation^12,13^. None of these studies quantified the required plume forcing and microplate rotation in detail. Large-scale numerical mantle flow simulations, however, suggest only a limited impact of basal shear tractions on East African plate kinematics^14,15^.

Here, we propose the hypothesis that Victoria rotation is controlled by the first-order geometry of adjacent rift arms, whose orientation is guided by the inherited distribution of mechanically stronger and weaker lithospheric domains. This hypothesis is inspired by previous observations of rotating oceanic microplates between two spreading centers, for which edge-driven microplate kinematics were proposed^16^, during which drag of the surrounding plates on the microplate’s edges drives rotation around a vertical axis. These fast-rotating^16,17^, short-lived (~5–10 My^18^) accreting oceanic microplates are bounded by weak overlapping ridge segments and by stronger coherent young oceanic lithosphere in the overall plate-boundary direction^19,20^. In East Africa, the plate-boundary configuration is similar, albeit at a much larger scale (Fig. 1b): the curved Eastern and Western Branches of the EARS delimiting Victoria run orthogonal to the regional relative extension direction and follow lithospheric suture zones formed during several Proterozoic orogenies^21–24^ (dashed areas in Fig. 1b). At the Tanzania divergence, the Eastern Branch separates into three strands that, in part, die out against the strong lithosphere of the Archean Tanzania craton^25,26^. Similarly, in northern Uganda, the Western Branch terminates at Precambrian fabric, the NW-trending Aswa Shear Zone^27^. The Aswa Shear Zone is in proximity to the location of a WNW–ESE trending, failed, early Cretaceous rift basin that is also traversed by the Eastern Branch^28^. This possibly polyphase rift^29^, which initiated the Turkana depression and the Anza graben^30^, significantly thinned the continental crust^31^. After thermal equilibration of the thinned radiogenic crust and the lithosphere, this failed rift now constitutes a mechanically strong region that is thought to affect the progression of the Main Ethiopian Rift and the Kenya rift approaching from the north and south, respectively^32,33^.

Although rotating microplates have been observed as specific modes of rift or ridge segment interaction in analog and numerical models^17,20,32,34–36^, it is unknown thus far whether the edge-driven mechanism^16^ applies to the rotation of large continental blocks like Victoria. In nature, in contrast to oceanic microplates, their continental counterparts generally evolve within complex inherited structures associated with previous episodes of mountain building or rifting^22,37–40^, and it is unclear how these anisotropies affect microplate rotation. In this study, we first use generic 3D numerical models to confirm edge-driven rotation of a Victoria-scale continental microplate, and to determine first- and second-order controls on such microplate rotation. Second, we demonstrate the applicability of the edge-driven mechanism to Victoria, in particular, by comparing the results of an EARS-specific numerical model to regional surface motions predicted from GNSS observations^3^. Finally, we compare the model-derived tectonic regime and stress field orientations with the respective EARS observations, providing a unifying perspective on plate kinematics and proposed stress sources of the EARS.

## Results

### Numerical model setup

**Fig. 2:**
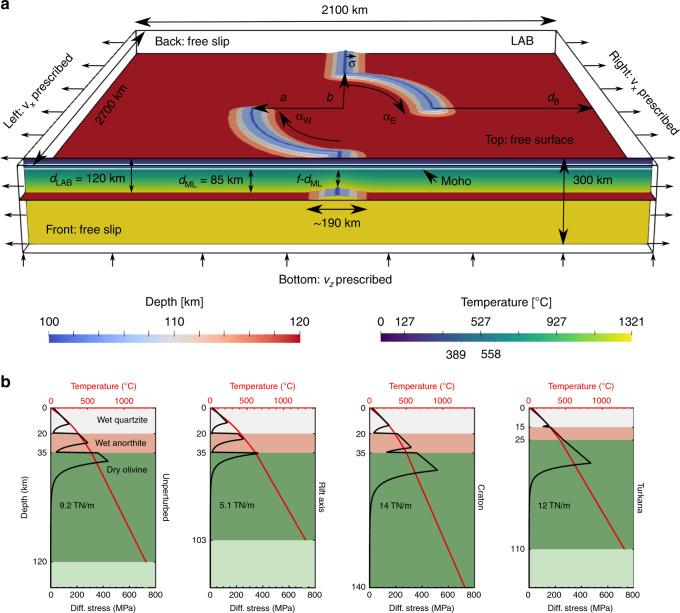
Generic mobile belt configuration model setup and lithospheric strength profiles. **a** A raised Lithosphere–Asthenosphere Boundary (LAB, depth shown in blue-to-red colors) is used to mimic the mobile belts. The perturbed depth of the LAB is constructed using a Gaussian distribution with standard deviation *σ* = 50 km and amplitude (thinning factor) *f* = 0.8. The lateral distribution of the perturbation can be divided into straight segments extending from the front and back domain boundaries and the curved segments that follow the outline of an ellipse centered at the midpoint of the domain, and can thus be characterized by the minor and major axes of the ellipse *a* = 375 km and *b* = 750 km, and their opening angles *α*_W_ = 90° and *α*_E_ = 90°. The minimum distance of the curved segments to the east and west boundaries is *d*_B_ = 675 km. Prescribed velocities are ${v}_{x}=\frac{1}{2}\cdot 5\,{\rm{mm}}\ {{\rm{yr}}}^{-1}$ in the reference case. The vertical slice shows the initial temperature field (which includes the same perturbation) together with the laterally homogeneous crustal layers outlined in white. Temperatures at the upper/lower crust and Moho interfaces are indicated below the temperature scale bar. Top and bottom temperatures are fixed at 273 and 1594 K, respectively. **b** Lithospheric strength and temperature profiles at representative locations for a lithostatic pressure and background strain rate determined by the velocity boundary conditions of  ~ 8 × 10^−17^ s^−1^.

We investigate the factors controlling generic and Victoria continental microplate rotation with the finite-element software ASPECT^41–44^. Our increasingly complex 3D model setups range from a generic, abstracted geometry of two arcuate rift branches within an otherwise homogeneous lithosphere to models specifically tailored to the EARS. The cuboid model domain of 2100 × 2700 × 300 km is initially filled with viscoplastic layers of the upper crust, lower crust, lithospheric mantle, and asthenosphere (Fig. 2; Supplementary Table 1), and a steady-state continental geotherm combined with an adiabatic mantle temperature profile. Mobile belts are represented by a raised Lithosphere–Asthenosphere Boundary (LAB); here deformation begins to localize under the outward velocity prescribed on the east and west boundaries. The configuration of the belts is characterized by the parameters defined in Fig. 2a.

### Generic mobile belt configuration models

The generic reference model (Fig. 2a) that forms the basis of our exploration of controls on microplate rotation includes an initial thinning of the lithosphere of  17 km along the outline of an ellipse with a minor axis *a* = 375 km and a major axis *b* = 750 km, comparable with the scale and geometry of the EARS as evident from the ellipse axes in Fig. 1b. The extent of the rift arm seed *α* is 90°. Due to initial thermal equilibration, the raised LAB reduces the strength of the lithosphere, which is a simplified approach to mimic the inherited weakness of mobile belts. We run the model for 10 My of model time while applying an extension velocity *v*_*x*_ of 2.5 mm yr^−1^ on both the left and right boundary, similar to what has been suggested for the EARS (see “Methods”).

**Fig. 3:**
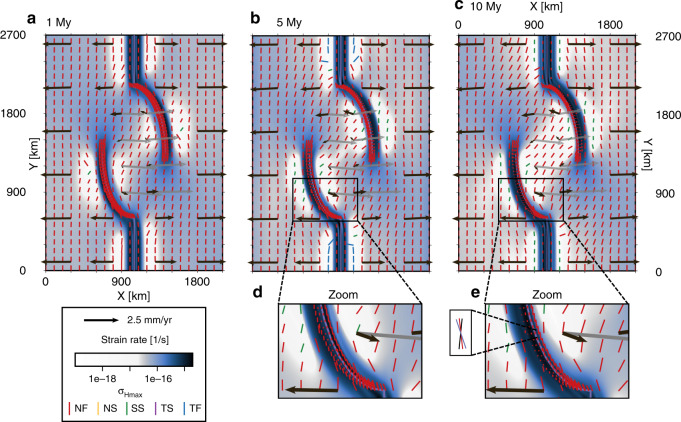
Generic mobile belt configuration reference model *Ellipse 90*^∘^ results. **a**–**e** Depicted are the strain rate field, maximum horizontal compressive stress ${\sigma }_{{\rm{Hmax}}}$ colored according to the tectonic regime, and the velocity vectors at 3-km depth at 1, 5, and 10 My. High-resolution ${\sigma }_{{\rm{Hmax}}}$ directions are plotted in an area of 100 km around the mobile belt axes if the strain rate exceeds 1 × 10^−16^ s^−1^. Black vectors are plotted in the model frame of motion, while the dark-gray vectors represent the microplate motion in a Nubia-fixed frame. Black and gray vectors are scaled equally. Panels **d** and **e** show a zoom-in of the oblique rift section in the boxed area indicated in **b** and **c**, respectively. The inset in **e** illustrates characteristic local deviations from far-field kinematics by comparing ${\sigma }_{{\rm{Hmax}}}$ (red), rift trend (purple), and the orthogonal of the local extension direction (black). NF normal faulting, NS normal and strike–slip faulting, SS strike–slip faulting, TS thrust and strike–slip faulting, TF thrust faulting.

**Fig. 4:**
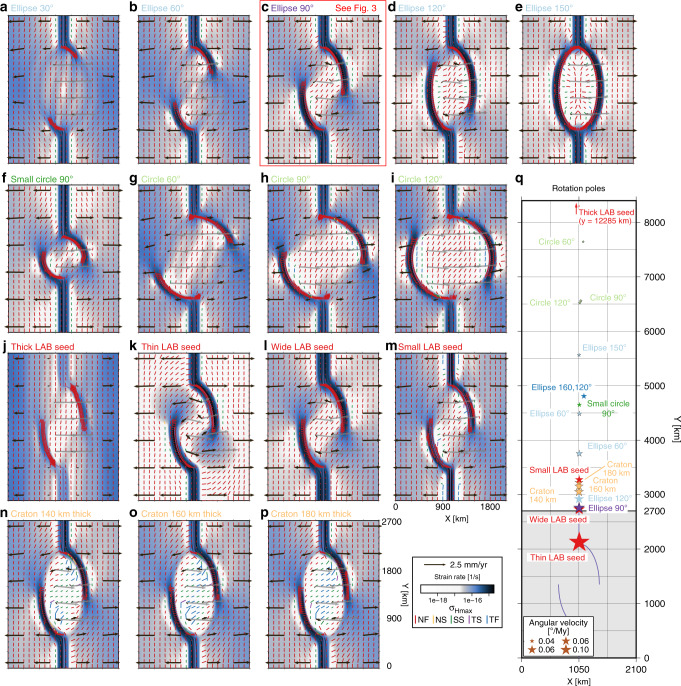
Controlling factors of the generic mobile belt configuration model. Depicted are the strain rate field, maximum horizontal compressive stress ${\sigma }_{{\rm{Hmax}}}$, and the velocity vectors at 3-km depth and 10 My for the generic models in **a**–**p**, together with the respective microplate rotation poles relative to the western plate in **q**. Black vectors in **a**–**p** are plotted in the model frame of motion, while the dark-gray vectors represent the microplate motion in a Nubia-fixed frame. Black and gray vectors are scaled equally. A red box indicates the reference model detailed in Fig. 3. Details on each of the models are given in Table 1. NF normal faulting, NS normal and strike–slip faulting, SS strike–slip faulting, TS thrust and strike–slip faulting, TF thrust faulting.

Figure 3 presents several snapshots of the reference model evolution. Deformation localizes along mobile belts, and as a consequence, the area inside the rift branches behaves progressively more rigidly. The velocity field shows E–W motion of the major plates (as dictated by the boundary conditions), with some loss of speed toward the rift due to internal deformation of the plates. Within the area spanned by the rift arms, the velocity field shows a rotational pattern around the center of the microplate. This motion is described by an angular velocity of 0.1021° My^−1^ around the vertical rotation axis passing through the point (*x*,*y*) = (1053, 2758) km (in a western-plate fixed frame, Fig. 4). As expected for this symmetric setup, the pole is very close to the central *x* coordinate (*x* = 1050 km). It is worth noting that a mirrored mobile belt geometry (such that the rift branch coming from the north is deflected west and the southern branch east) produces the same, but clockwise, rotation of the microplate.

Within the two major plates, the maximum horizontal compressive stress is predominantly N–S oriented. Inside the rifts, however, the local extension direction and ${\sigma }_{{\rm{Hmax}}}$ deviate from the far-field extension and corresponding stress direction. In the curved rift segments, the stress orients itself at an angle to both the extension direction and the rift axis (Fig. 3e), a process that has been described in previous analog and numerical studies^45–47^ of less complex setups. In front of the rift tips, ${\sigma }_{{\rm{Hmax}}}$ rotates such that it aligns with the outline of the ellipsoidal microplate.

We find that rift-parallel motion is the largest along the most oblique segments of the rift, up to 30 km over the full 10 My. The rotation of Victoria also results in block-parallel motion along the more diffuse microplate boundaries, but only up to 22 km. The curved rift segments each accommodate between  ~ 19 and 32 km of E–W extension in 10 My (the total applied extension is 50 km). Figure 3 also shows that Nubia and Somalia plate motions outside the rift sections are E–W, while in the more coupled areas, the velocity field is deflected in the direction of rotation. Note that the entire motion field is point-symmetric around the center of the block (gray arrows in Fig. 3c).

In the following, we assess the impact of possible controls on the microplate rotation by varying, with respect to the above reference model, first the lateral distribution of weaknesses in terms of their extent (opening angle *α*), their offset (ellipse minor axis *a*), and their symmetry (opening angles *α*_W_ and *α*_E_), then the lithospheric strength in terms of initial LAB-thinning amplitude (thinning factor *f*) and width (standard deviation *σ*), and finally the thickness of the microplate.

Varying the initial extent of the mobile belts between 30° and 150° in steps of 30° predicts the derived rotation poles in Table 1 and Fig. 4q, while velocity, stress, and strain rate fields after 10 My are shown in Fig. 4a–e. The predicted angular velocity is the highest for an opening angle of 90°, and decreases with deviation from this optimum angle, accompanied by a northward shift of the rotation pole. These changes are not symmetric around 90°, however. For an extent of 30°, the strain rate field shows an ellipsoidal pattern of deformation, as well as two additional high-strain rate branches directly crossing the potential microplate area. From 90° onward, the ellipsoidal area between the branches is seen to behave as a coherent block, which is also reflected in the low root-mean-square residual distance (RMSD) of the pole location (Table 1). For an extent of 150°, the rift arms connect to the straight rift segments to form a full ellipse, in effect prohibiting motion of the microplate. In all actively deforming areas, the tectonic stress regime is extensional, while in low strain rate areas, some strike–slip regimes result.

**Table 1:** Predicted rotation poles relative to the western plate after 10 My for different controlling parameters of the generic model setup.

Model name	Extent	Minor	Major	*d*_B_	Thinning	*σ*	Craton	Pole	RMSD	Angular	Fig.
	*α*_W_, *α*_E_	axis *a*	axis *b*		factor *f*		LAB	location		velocity	
	[°]	[km]	[km]	[km]	[−]	[km]	[km]	[km, km]	[km]	[°My^−1^]	
Ellipse 30°	*30*	375	750	675	0.8	50	120	(1057, 4487)	131	0.0454	4a
Ellipse 60°	*60*	375	750	675	0.8	50	120	(1055, 3755)	57	0.0597	4b
*Ellipse 90°	*90*	375	750	675	0.8	50	120	(1053, 2758)	42	0.1021	4c
Ellipse 120°	*120*	375	750	675	0.8	50	120	(1051, 2917)	27	0.0914	4d
Ellipse 150°	*150*	375	750	675	0.8	50	120	(1051, 5566)	23	0.0340	4e
Small circle 90°	90	375	375	675	0.8	50	120	(1057, 4650)	38	0.0435	4 f
Circle 60°	*60*	*750*	750	300	0.8	50	120	(1128, 7647)	142	0.0228	4g
Circle 90°	90	*750*	750	300	0.8	50	120	(1085, 6562)	52	0.0276	4h
Circle 120°	*120*	*750*	750	300	0.8	50	120	(1063, 6521)	36	0.0277	4i
Ellipse 160,120°	*160*,* 120*	375	750	675	0.8	50	120	(1141, 4808)	32	0.0523	–
Thick LAB seed	90	375	750	675	*0.9*	50	120	(1053, 12285)	64	0.0131	4j
Thin LAB seed	90	375	750	675	*0.7*	50	120	(1055, 2125)	43	0.1871	4k
Wide LAB seed	90	375	750	675	0.8	*75*	120	(1054, 2732)	42	0.1041	4l
Small LAB seed	90	375	750	675	0.8	*25*	120	(1054, 3275)	50	0.0745	4m
Craton 140 thick	90	375	750	675	0.8	50	*140*	(1050, 3051)	7	0.0843	4n
Craton 160 thick	90	375	750	675	0.8	50	*160*	(1050, 3153)	1	0.0795	4o
Craton 180 thick	90	375	750	675	0.8	50	*180*	(1050, 3223)	1	0.0766	4p

*RMSD* root-mean-square residual distance.

Italic values indicate the parameters varied with respect to the reference model *Ellipse 90*° (*).

As shown in Table 1 and Fig. 4c and h, extending the width between the arms (increasing minor axis *a*, circle models) reduces the rotation velocity and increases the distance of the pole to the center of the domain. Decreasing the major axis *b* to 375 km to obtain a smaller circular configuration leads to a smaller reduction of microplate rotation (Fig. 4f).

As for *a* = 375 km, for circular mobile belt configurations (*a* = 750 km), rotation velocity drops when the opening angle *α* is smaller than 90° (Table 1 and Fig. 4g–i). However, changes in angular velocity and pole location are less evident for opening angles  >90°. Strike–slip regimes develop in the most oblique segments of the circular rifts with *α* ≥ 90°.

EARS-like asymmetric opening angles of *α*_W_ = 160° and *α*_E_ = 120° reduce the angular velocity of the microplate by a factor  ~ 2 (Table 1 and Fig. 4q). The rotation pole is displaced northward and, moreover, to the east. This shift is due to the higher coupling along the eastern side of the microplate compared with the completely decoupled western side.

Varying the thinning factor *f* and the rift axis normal extent of the seed *σ* as detailed in Table 1 and Fig. 4j–m shows that a thinning factor that is too small (model thick LAB seed) does not localize deformation in the rift arms. A thinner LAB, however, enhances localization, leading to an almost doubled angular velocity and rigid plates. A doubled mobile belt width has an insignificant effect on the model results, while a smaller *σ* reduces the rotation of the microplate by up to ~25%.

Increased strength of the microplate due to a thicker lithosphere (140, 160, or 180 km) for a fixed crustal thickness induces more plate-like behavior of the microplate (i.e., lower internal strain rates in Fig. 4n–p and small RMSD in Table 1). However, rotation velocity decreases with increased thickness. Perhaps, the velocity decrease is caused by the interaction of the increasingly thick cratonic root with the mobile belts, reducing the initial LAB perturbation. The regions undergoing strike–slip faulting in the most oblique rift sections grow with craton thickness. Within the interior of the craton, the tectonic regime transitions from strike–slip to thrust faulting.

### East African Rift System-specific mobile belt configuration models

**Fig. 5:**
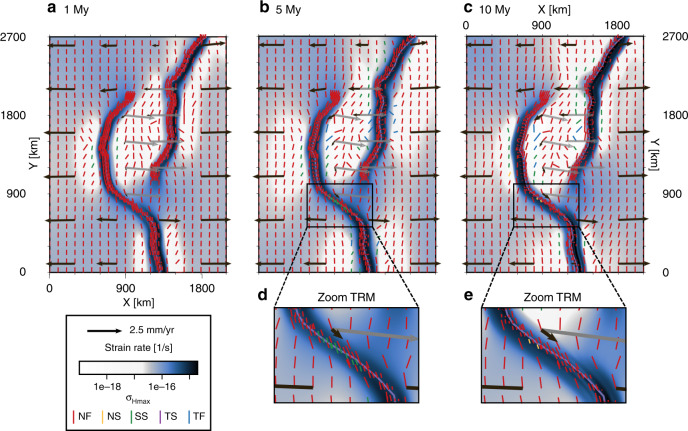
East African Rift System-specific mobile belt configuration *EARS* model results. **a**–**e** Depicted are the strain rate field, maximum horizontal compressive stress ${\sigma }_{{\rm{Hmax}}}$ colored according to the tectonic stress regime, and the velocity vectors at 3-km depth for the East African Rift System initial mobile belt configuration at 1, 5, and 10 My. Black vectors are plotted in the model frame of motion, while the dark-gray vectors represent the microplate motion in a Nubia-fixed frame. Black and gray vectors are scaled equally. Panels **d** and **e** show a zoom-in of the oblique rift section in the boxed area indicated in **b** and **c**, respectively. NF normal faulting, NS normal and strike–slip faulting, SS strike–slip faulting, TS thrust and strike–slip faulting, TF thrust faulting.

In this second set of models, we account for first-order rift geometry of the EARS, including the asymmetry of the Western and Eastern Branches and the obliquity of the straight rift segments. As Fig. 5 shows, the realistic EARS geometry does not change the validity of the edge-driven microplate mechanism, but does affect the tectonic regime of specific areas. For example, within the Tanganyika–Rukwa–Malawi (TRM) segment, the tectonic regime transitions from normal faulting at 1 My, to strike–slip at 5 My, and oblique normal slip at 10 My. The other rift segments exhibit normal faulting only. Consistent with the generic model, the normal faults in the rift form at half the angle between the mobile belt trend and the local extension direction (which deviates from E–W). Over time, both the plate-like area and the rotation velocity increase (summarized in Table 2).

**Table 2:** Predicted rotation poles relative to Nubia at 5 and 10 My for different lithospheric strength configurations and prescribed extension velocities for the East African Rift System-specific mobile belt configuration model setup.

Model name	Time	${v}_{x}^{{\rm{W}}}$,${v}_{x}^{{\rm{E}}}$	Tanzania craton	Turkana depression	Pole location	RMSD	Angular velocity	Fig.
	[My]	[mm yr^−1^]	[km]	[km, km]	[km, km]	[km]	[°My^−1^]	
*EARS	5	−2.5, 2.5	120	20,15	(1211, 3148)	4	0.0651	
	10				(1203, 3197)	4	0.0772	6a
Craton	5	−2.5, 2.5	*140*	20,15	(1234, 3336)	5	0.0875	
	10				(1244, 3183)	4	0.0964	6b
Anza	5	−2.5, 2.5	120	*15,10*	(1166, 3303)	8	0.0698	
	10				(1178, 2887)	4	0.0866	6c
N–S vel. decrease	5	$\pm \frac{{v}_{{\rm{e}}}+\Omega (y-{y}_{{\rm{c}}})}{{2000}}$	120	20,15	(1137, 3634)	13	0.0468	
	10				(1138, 3303)	12	0.0542	6d
N–S vel. craton	5	$\pm \frac{{v}_{{\rm{e}}}+\Omega (y-{y}_{{\rm{c}}})}{{2000}}$	*140*	20,15	(1176, 3395)	6	0.0643	
	10				(1190, 3349)	8	0.0637	6e
N–S vel. craton Anza	5	$\pm \frac{{v}_{{\rm{e}}}+\Omega (y-{y}_{{\rm{c}}})}{{2000}}$	*140*	*15,10*	(1153, 3233)	6	0.0686	
	10				(1165, 3076)	5	0.0708	6f

*RMSD* root-mean-square residual distance.

Italic values indicate the parameters varied with respect to the first model *EARS* (*). *v*_e_ = 0.3983 m yr^−1^, *Ω* = 9.4295 × 10^−5^ m yr^−1^ km^−1^, *y*_c_ = 1350 km.

Predicted motion of the Nubian plate is E–W, except for some southward component near the northern rift tip; some northward movement is predicted for Somalia. Areas located within the microplate and to the east of the TRM segment have a significant N–S component of motion, in accordance with the counterclockwise rotation of Victoria. Along the TRM segment and the oblique northern part of the Eastern Branch, rift-parallel motion is the largest, up to 26 km over 10 My.

**Fig. 6:**
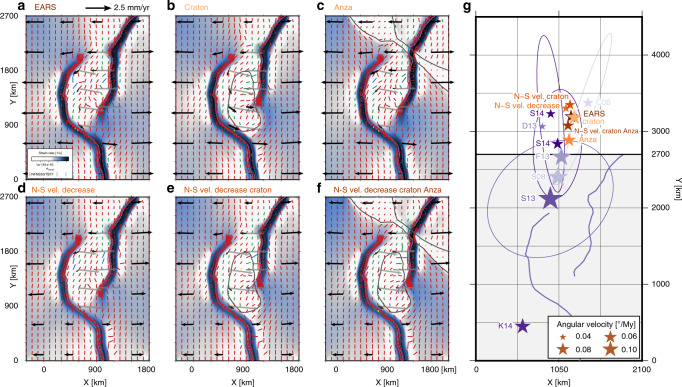
Controlling factors of the East African Rift System-specific mobile belt configuration model. **a**–**f** Depicted are the strain rate field, maximum horizontal compressive stress ${\sigma }_{{\rm{Hmax}}}$ colored according to the tectonic stress regime, and the velocity vectors at 3-km depth and 10 My for the East African Rift System initial mobile belt configuration models. Black vectors are plotted in the model frame of motion, while the dark-gray vectors represent the microplate motion in a Nubia-fixed frame. Black and gray vectors are scaled equally. Tanzania craton and Turkana depression outlines are plotted in gray when used in the initial conditions. **g** In brown, the microplate rotation poles relative to the Nubian plate. Rotations poles from geodetic data inversion are included in purple, for references see the caption of Fig. 1a. The model domain is indicated in gray, and the  mobile belt axes in purple. Details on each of the models are given in Table 2. NF normal faulting, NS normal and strike–slip faulting, SS strike–slip faulting, TS thrust and strike–slip faulting, TF thrust faulting.

Inclusion of the Tanzania craton that is partially traversed by the Eastern Branch prohibits substantial localization of the modeled Eastern Branch in the cratonic area (Fig. 6b), although increased strain rates are seen compared to the rest of the craton. Deformation is diverted around the eastern edge of the craton, and the maximum horizontal stress aligns with the craton edge. Increased transmission of plate motion to the microplate through the strong craton leads to higher angular velocities and a slight displacement of the pole to the east (Table 2). A similar effect is seen when the Turkana depression is included through a local thinning of the upper and lower crustal layers to 15 and 10 km, respectively. The depression locally deflects the stress field to align with the depression, except in front of the Western Branch tip, where stress rotates to become orthogonal to the depression. Transmission of motion is larger in the north, bringing the larger rotation pole westward and closer to the microplate (Fig. 6g).

A gradient in the prescribed velocity (the domain-boundary orthogonal component of the velocity computed from the Nubia–Somalia Euler pole^3^; Table 1; Fig. 6d) to mimic the N–S relative velocity decrease of the Somalian plate (Fig. 1a) decreases the strain rate in the southern parts of the system. This prolongs the existence and extent of the strike–slip regime in the TRM segment and reduces the angular velocity. Due to the reduced velocity in the south, the addition of the strong Tanzania craton (Fig. 6d) affects the results less than in the case of uniform boundary conditions, although the pole varies less between 5 and 10 My. Together with the stronger Turkana depression (Fig. 6f), a Victoria microplate rotation of 0.0708° My^−1^ around a pole at (1165, 3076) km is found. This latter model, the most complex model, matches best with the most recent Victoria rotation^3^ of 0.0740° My^−1^ at (1032, 2837) km in our coordinate system, as illustrated by Fig. 6g. All models with an EARS mobile belt configuration predict rotation poles that plot within the area spanned by geodetically derived Euler poles^1,3,6^.

In summary, our results show that with ongoing localization over time, the relative instantaneous rotation pole describing the microplate rotation moves closer to the microplate. Contemporaneously, the residuals in pole location decrease, signifying a more rigid behavior of the microplate. Larger extent of the pre-existing weaknesses and a thicker microplate also increase the plate-like behavior. Only highly localized models have the instantaneous rotation pole at, or inside, the microplate boundaries; most of them lie less than 1000 km north of the microplate. Stresses in high-strain rate regions indicate tension and consequently normal faulting, except when the strength of the craton exceeds that of the major plates’ lithosphere or under highly oblique, faster extension. In front of the rift tips, the maximum compressive stress aligns with the edges of the microplate. Despite the overall extensional setting, we find that rigid areas may exhibit strike–slip and even thrust-faulting regimes.

## Discussion

**Fig. 7:**
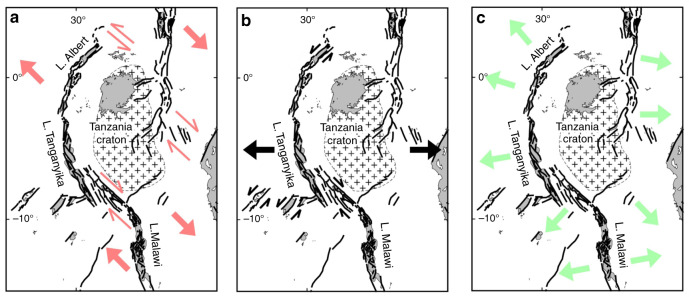
Previously proposed kinematic opening models of the East African Rift System. **a** NW–SE regional extension with large strike–slip motion on NW-striking structures such as the Tanganyika–Rukwa–Malawi segment^25^. **b** Regional E–W extension leading to a minor strike–slip component on oblique segments^53^. **c** Regional E–W extension with predominantly normal faulting suborthogonal to the rift segments^54^.

**Fig. 8:**
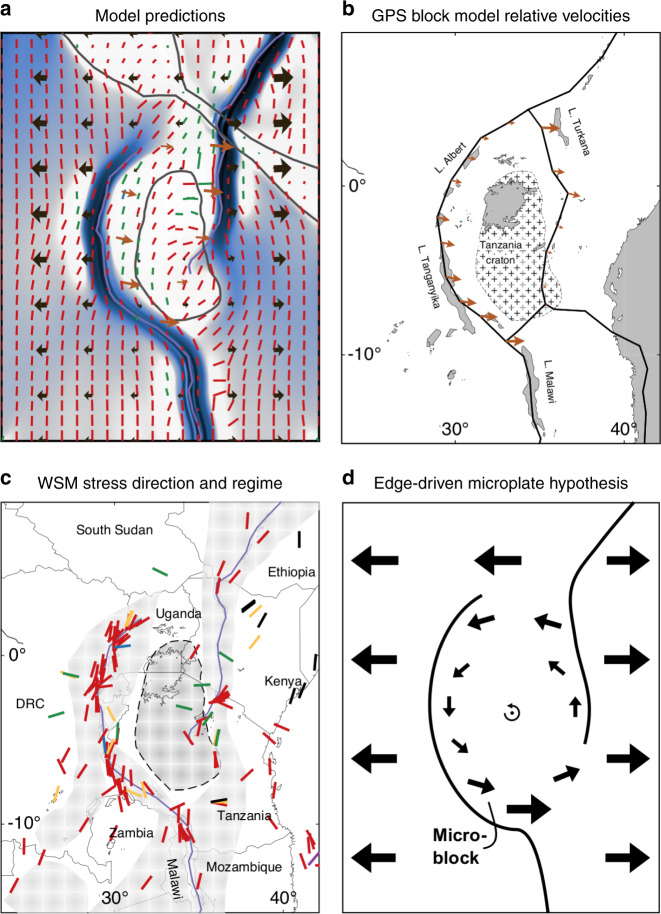
Summary plot of East African Rift System modeling, data, and interpretation. **a** Results of model NS vel. craton Anza, including ${\sigma }_{{\rm{Hmax}}}$, and indicating in black the absolute velocity field and in orange the relative motion of Victoria with respect to the Nubia plate along the Western Branch (at 100 km from the original  mobile belt axis) and the relative motion of the Somalia plate w.r.t. Victoria along the eastern plate boundary (as in panel **b**). **b** Kinematic block model prediction of Victoria microplate rotation^3^. Relative velocity vectors in orange represent the motion of the plate to the east of the plate boundary w.r.t. the plate to the west of the boundary. **c**${\sigma }_{{\rm{Hmax}}}$ directions from the World Stress Map^55^ (WSM), including all data types and qualities, colored according to tectonic regime as in Figs. 3–6. Note that ${\sigma }_{{\rm{Hmax}}}$ bars of the WSM have a uniform length that does not represent their magnitude. **d** Our schematic representation of the edge-driven Victoria microplate rotation due to transmittance of the motion of the major plates.

We first discuss our generic model results in light of previous modeling studies. Then we examine our EARS-specific findings and their implications for our understanding (Fig. 7) of stress patterns and kinematics of the present-day EARS (Fig. 8).

The generic 3D numerical models presented demonstrate how a large continental microplate can rotate under homogeneous far-field extension through the edge-driven mechanism. This rotation is predominantly controlled by the length and obliquity of the stronger microplate edges where the motion of the major plates is transmitted to the microplate, that of the diverging segments facilitating the rotation, and the absolute distance between the segments.

Our models demonstrate that the edge-driven oceanic microplate model^16^ is also a valid driving mechanism for continental microplate rotation in the sense that the drag of the bounding plates generates the rotation. Similar to the oceanic microplate examples^16^, our relative instantaneous poles are not located at or even close to the rift tips, as suggested for the idealized edge-driven oceanic microplate model^16^. With the poles several hundred kilometers away from the Victoria microplate, this difference could indicate slip between the plates or a contribution from viscous coupling to mantle flow^16^. The orthogonal geometric relation between the line connecting the instantaneous rotation poles and the regional extension direction in the idealized edge-driven model^16^ also does not hold when the prescribed velocity varies along the model boundaries.

On a smaller scale than continental and oceanic microplates, microblock rotation due to drag of the bounding blocks was observed in 2D visco–elasto-plastic spring models^17^, in which ~30–70 km-sized microblocks were captured by offset fault segments and subsequently rotated with ongoing fault opening. Comparable with our findings, the direction of rotation depended on the kinematics of the transfer zone: in the case of a right-lateral step, rotation was counterclockwise and vice versa.

The offset between interacting mid-oceanic ridge or continental rift segments has been shown to be a crucial component in the formation of microplates. Through analog modeling, it was found that the ratio of the total length of the interacting segments to the initial offset determines whether a transform develops (for a small ratio) or an overlapping spreading center (OSC, for a large ratio), a precursor of oceanic microplate formation, is generated^48^. Crustal-scale 3D numerical models of continental rift interaction^35^ showed an overlapping, propagating, but non-linking mode for intermediate brittle–ductile coupling and larger offsets (≥4 times the upper crustal thickness). Growing rotating microplates between OSCs and nonrotating microplates between parallel ridges also form in the crustal-scale models for large initial offsets (≥60 km) and slow-healing rates^49^. Similar kinematics are found for oblique pre-existing weak or strong regions: a northwest trending oblique rift seed and coupled crust and lithospheric mantle produced a counterclockwise rotating OSC in 3D numerical models^36^. In another numerical study^32^, counterclockwise rotating continental microplates formed in the case of left-laterally deflected propagating rifts as a consequence of an oblique, NW-trending strong inherited structure.

Based on the above modeling results of previous studies, it is evident that not only the segment offset, but also the strength and thickness of the lower crust, and thus the brittle–ductile/crust–mantle coupling, are important factors in switching between different modes of interaction of extensional segments. We have not explored the space spanned by the rheological and crustal thickness parameters, but instead used standard flow law parameters and crustal thicknesses representative of the region. Only for the smallest extents of the mobile belts or a small perturbation of the LAB, the rifts fail to localize sufficiently or directly crosscut the would-be microplate (Fig. 4).

There is consensus on the relationship between the inherited weakness of the mobile belts and the location of the curved, overlapping EARS segments^21,23,39,50^. Other modeling studies^26,35,49,51,52^ have also shown that two overlapping rift segments can form without pre-existing strength heterogeneities like mobile belts (so-called soft linkage). These overlapping segments would again set the stage for the edge-driven mechanism to generate rotation of the encompassed microplate.

Our EARS-specific models provide a good fit with kinematic models from geodetic data inversion. Figure 8a shows the predicted relative motion of the Victoria microplate and the Somalia plate w.r.t. their respective eastern neighbors. The southward, resp. northward, increase in extension velocity and higher relative velocities in the northeast versus southwest match the trends in the predicted plate motions^3^ plotted in Fig. 8b. Also, for a specific latitude, extension is not equally distributed between the Eastern and Western Branch^1^. As such, the rotation pole describing Victoria’s predicted rotation ((1165, 3076) km and 0.0708° My^−1^) fits the pole obtained from GNSS observations^3^ ((1032, 2837) km and 0.0740° My^−1^) well within the confidence interval. Based on the generic modeling results, inclusion of the continuation of the Mozambique belt around the southeastern edge of Victoria (Fig. 1b), as well as variations in rift-normal extent of the belts, would probably improve the fit between our predicted pole and that found from block modeling.

Many authors investigating oceanic microplate rotation dismiss a mantle contribution to the rotation^20^ and even assign mantle drag to the resisting forces^56^. The Victoria microplate’s counterclockwise motion has, in contrast, been suggested to derive from mantle flow interacting with the Tanzania craton’s keel^1,12,13^. An earlier study^1^ argued that, since models of plume–craton interaction show focusing of mantle flow around the keel, and seismic anisotropy indicates NE-ward mantle flow underneath East Africa, this flow could act on the thickest, southern part of the Tanzania craton, inducing a rotation. However, the authors note that the WNW motion of Nubia predicted by the applied global hotspot frame does not match anisotropy directions, and thus, attribute these to plume-related flow. However, in more recent plate motion reference frames^57^, absolute Nubia plate motion is NNE (Fig. 1a), agreeing with the cited seismic anisotropy. Also, there is some disagreement on the distribution of low seismic velocity anomalies seen in the East African upper mantle. For example, whereas some studies^58,59^ advocate for one East African superplume, others^60^ distinguish separate lower and upper mantle plumes, or consider a lower mantle plume splitting in the upper mantle^61^, complicating the interpretation of seismic anisotropy in terms of plume-induced flow.

Plume–craton interaction has been extensively investigated^12,13,62,63^ with 3D upper mantle box models. Counterclockwise rotation of a central cratonic block was obtained in several model configurations^13^, including models with a single-plume offset from the center and with two plumes impinging on the NE and SW corners of the craton. Localization of strain initially driven by the applied far-field extension was intensified by plume-related heat transport and channeling of plume material. The authors attribute the counterclockwise rotation to the distribution of plume forces interacting with the keel of the craton. At the same time, the plume head impingement in their models induces a configuration of strain localization and lithospheric weakness similar to our EARS models. It is hence not clear whether Victoria microplate rotation in their models is predominantly due to plume push or actually controlled by the edge-driven mechanism discussed above. This is not to say that plume-induced flow cannot contribute to microplate rotation; our models, notably those without a craton, however indicate that it is not required to generate such rotation. Numerical models^64^ did show that a plume will travel underneath the lithosphere to places of rheological contrast. Under slow extension, breakup occurred at the rheological contrast. In that sense, the heterogeneity in the lithosphere due to the presence of mobile belts in the EARS could attract a plume that will heat and raise the LAB, while both the heterogeneity and the plume help to localize the deformation. Therefore, whether crustal/lithospheric heterogeneity initially localizes deformation and attracts a plume, or lithospheric heterogeneity in itself is enough to localize deformation (as in this study), the basic requirement is still lithospheric heterogeneity under far-field extension.

Our EARS model that best matches geodetic constraints provides stress predictions at the large and intermediate scale that we can compare with observations of the present-day and paleo stress field. Figure 8a and c juxtaposes the present-day maximum horizontal stress directions of model N–S vel. craton Anza and the World Stress Map^55^ (WSM). In general, our predicted stress orientations agree well with the WSM: predominantly normal faulting regimes are seen that rotate along the curved Western Branch, but are directed at an angle to the rift trend in the more oblique sections. However, we do not reproduce the more E–W-oriented normal faulting seen in the Western Branch around a latitude of  −3°N. WSM data in the Rukwa rift area are scarce, but some oblique normal faulting is documented and compares very well to our model predictions. Inside the microplate, in the more rigid domains, we obtain a good match with ${\sigma }_{{\rm{Hmax}}}$ in strike–slip mode trending NNE along the Western Branch and E–W along the Eastern Branch. Also, the rotation of ${\sigma }_{{\rm{Hmax}}}$ west of the Tanzania divergence matches well.

The few occurrences of tectonic stress regimes not conducive to normal faulting seen in the WSM were also found through processing of focal mechanisms^65^, with strike–slip regimes speckled around the Western and Eastern Branches. The obtained box averages, however, only leave normal-to-oblique normal slip regimes, with the exception of strike–slip in the Mbeya area between Lakes Rukwa and Malawi. Focal mechanisms are local indicators of the stress field that can be affected by higher-order stress sources. For example, stress can locally be deflected by oblique fabrics, such as the foliation in the Ubende belt. This hypothesis was put forward for the Rukwa rift^53^: the strength anisotropy deriving from the foliation reorients the stress field such that the oblique rift does not exhibit the predicted oblique slip, but pure normal faulting. Analog modeling studies^66,67^ even show stress reorientation along single faults within an oblique rift segment. Since our initial model conditions cannot take into account small-scale fabric trends such as foliation, nor can we examine our results at individual-fault scale, it is not surprising that we do not obtain the small-scale stress variations obtained from in situ measurements. One important observation from our models is, however, that the regional ${\sigma }_{{\rm{Hmax}}}$ does not need to be oriented orthogonally to the velocity nor to the relative velocity, or parallel to the rift trend. Hence, direct comparison of kinematic models and stress indicators is not warranted. For example, good agreement in direction (≤20°) between model velocities from GNSS stations^6^ and focal mechanisms^65^ was found in the Albertine, Kivu, and northern Tanganyika rifts (Fig. 1b), but large deviations along the southern Tanganyika and Rukwa rifts^6^. This disagreement between velocity and stress directions is clearly demonstrated in our modeling (Fig. 8) and, as such, a mismatch between kinematic and stress directions does not mean the kinematic model fails to describe the deformation of the system under investigation.

The opening kinematics of the fault segments along the EARS branches have been the topic of a long-standing debate that can be represented by three kinematic models: based on the arcuate shape of specifically the Western Branch and local kinematic indicators, the EARS was initially interpreted as a strike–slip system with a relative NW–SE extension direction^25,68–71^ implying large strike–slip motion especially on the TRM and Aswa segments (Fig. 7a). In a second view, the EARS is considered an extensional system resulting from relative E–W regional extension, where the strike of obliquely oriented rift segments was guided by pre-existing fabrics of mobile belts^53,72–77^. In this view, oblique rifts like the TRM segment (Fig. 1b) are also expected to experience some degree of strike–slip motion (Fig. 7b). More recently, a modified version of this model was put forward^54,65^, in which deformation is purely extensional and orthogonal to the rift segments, including the TRM segment (Fig. 7c). This last opening model is informed by numerous available focal mechanisms indicating normal faulting that have been collected since the earlier models were suggested. As mentioned by these authors, the stress field may have changed over time, complicating the interpretation of fault–slip measurements in terms of regional extension direction. For example, quaternary clockwise rotations of the extension direction from ENE–WNW to NW–SE were inferred in the central Kenya rift^78^ and the Malawi rift^79,80^.

Based on present-day and inferred past regional extension directions and our model results, we can synthesize the three EARS opening models presented in Fig. 7. In an overall E–W- extending system of partly overlapping arcuate rift branches^53,72–77^, our edge-driven microplate model intrinsically leads to local WNW–ESE extension directions^25,68–71^. In the oblique rift sections, normal faulting thus occurs at a small angle to the trend of the rift and pre-existing weaknesses with few strike–slip occurrences (e.g., Fig. 8b; ref. ^65^). Nevertheless, the rift-parallel and -orthogonal motion is predominantly accommodated by normal faulting^54,65,81^. Small-scale sources such as basement fabric locally deflect the stress field such that even the nonoverlapping, oblique TRM section deforms under a mostly normal-faulting regime^53^.

In conclusion, our suite of generic models demonstrates that the spatial distribution of lithospheric weakness (i.e., extent and distance) exerts a first-order control on microplate rotation. The strength (i.e., thickness) of the microplate and the geometry of the weak zones in the plane orthogonal to the rift axis are only of second-order control. For model geometries with right-lateral rift branch stepovers, we find a counterclockwise rotation of the microplate, which generates a clockwise shift of the local extension direction along the overlapping rift branches.

Moreover, we provide a mechanical explanation for the counterclockwise rotation of the continental Victoria microplate in the Nubia–Somalia divergent plate boundary. The edge-driven rotation stems from shear of the major plates along the northwest and southeast corners of the microplate, where both a strong failed rift and cratonic lithosphere transmit this motion and divert propagation of the rift branches surrounding the Victoria microplate. These overlapping rift branches following the weak Proterozoic suture zones around the craton facilitate the rotation. Under regional  ~E–W extension, this modeled rotation results in local extension directions that strike more WNW–ESE. Together with the oblique orientation of the pre-existing weaknesses, the rotation leads to predominantly normal faulting oblique to the regional and local extension direction. In the most oblique (~45°) section of the Western Branch, the Tanganyika–Rukwa–Malawi segment, the rotation can produce transient strike–slip faulting, although comparison to stress observations suggests a local stress reorientation, possibly due to inherited mechanical anisotropy.

## Methods

### Governing equations

We use the open-source, massively parallel, finite-element code ASPECT^41–44^ to solve the extended Boussinesq equations of momentum, mass, and energy (assuming an infinite Prandtl number) combined with advection equations for each Eulerian compositional field *c*_*i*_

1 $$-{\boldsymbol{\nabla }}\cdot \left(2\eta \dot{\epsilon }\right)+{\boldsymbol{\nabla }}P=\rho {\bf{g}}$$

2 $${\boldsymbol{\nabla }}\cdot {\bf{v}}=0$$

3 $$\bar{\rho }{c}_{P}	\left(\frac{\partial T}{\partial t}+{\bf{v}}\cdot {\boldsymbol{\nabla }}T\right)-{\boldsymbol{\nabla }}\cdot (k+\nu ){\boldsymbol{\nabla }}T=\bar{\rho }H\\ +	 \, 2\eta \dot{\epsilon }:\dot{\epsilon }\\ +	 \, \alpha T({\bf{v}}\cdot {\boldsymbol{\nabla }}P)$$

4 $$\frac{\partial {c}_{i}}{\partial t}+{\bf{v}}\cdot {\boldsymbol{\nabla }}{c}_{i}-{\boldsymbol{\nabla }}\cdot \nu {\boldsymbol{\nabla }}{c}_{i}=0,$$

where $\dot{\epsilon }$ is the deviator of the strain rate tensor $\frac{1}{2}(\nabla {\bf{v}}+{(\nabla {\bf{v}})}^{T})$, density $\rho ={\rho }_{0}(1-\alpha (T-\bar{T}))$ with $\bar{T}$ the adiabatic reference temperature, $\bar{\rho }$ is the adiabatic reference density, and *ν* is the artificial diffusion. All other symbols are defined in Supplementary Table 1.

### Model domain

The governing equations are solved on a rectangular cuboid domain of 2100-km length in the *x*-direction and *y*- and *z*-dimensions of 2700 and 300 km, respectively (see outline in Fig. 1a and the actual domain in Fig. 2).

The domain is at start-up variably discretized in depth, leading to a lithospheric resolution of 9.375 km, while below 160-km depth, resolution is 18.75 km. The mesh resolution is kept constant over time, while mesh nodes can be displaced vertically in response to the free surface. For optimal use of the second-order finite elements, visualization of the solution is performed on a mesh of twice this resolution. Each model run requires about 8 h of run time using 800 processes.

### Initial conditions

The model domain is filled with four compositional layers—upper crust, lower crust, mantle lithosphere, and sublithospheric mantle—that are individually perturbed to initiate rifting or to create areas of different rheological strength (e.g., representing the thick, strong Tanzania craton or the weak mobile belts). The reference Moho is set at 35 km, which lies within the inferred range of our study area^83,84^. We choose the unperturbed Lithosphere–Asthenosphere Boundary (LAB) to lie at 120-km depth, loosely based on several lithospheric thickness maps^84^ and within the range of the global thermal model TC1^85^. The initial temperature distribution in the lithosphere follows a steady-state geotherm^86^ that considers the local thickness of the compositional layers and their material properties (i.e., density, thermal conductivity, and radioactive heating) along 1D depth profiles (i.e., solutions of $T^{\prime\prime} (d)=-\frac{H}{k}$). The LAB is defined as a specific isotherm (1576 K), below which a mantle adiabat (${T}_{a}^{\prime}(d)=\frac{\alpha | g| T}{{C}_{p}}$) is assumed. The resulting surface heat flow (58–66 mW m^−2^ for model real N–S vel. decrease craton Anza at start-up) matches the overall magnitudes and trends of heat flow measurements (15–75 mW m^−2^) and predictions (20–185 mW m^−2^)^87^.

Present-day data point to mobile belts as regions of smaller thermal^85^ and elastic^88^ thickness where strain localizes during the supercontinent cycle^89^. LPO-induced mechanical anisotropy of the lithospheric mantle could also contribute to the preferred reactivation of these highly deformed belts^37^. We capture all possible sources of the weakness of mobile belts (e.g., foliation and higher temperatures) and/or the impingement of plume material at such strength contrasts^64^ by representing the mobile belts through perturbations of the LAB, mimicking a small amount of lithospheric thinning. The perturbations are specified through a Gaussian distribution of thinning factor *f* around line segments representing the belt axes (see Fig. 2 for the definition of parameters *f*, *α*, *a*, and *b* that define the mobile belt geometry). Under the assumption of thermal steady state, the thinner mantle lithosphere in the belts leads to higher temperatures in the lithosphere, which in turn decrease its strength (compare the first two strength profiles in Fig. 2). The integrated strength in the mobile belts is  5.1 TN m^−1^, which is in line with the estimates^90^ of 3–5 TN m^−1^ for the force available for general continental extension.

The more complex EARS-specific models include mobile belt and Turkana depression geometries that are digitized from Fig. 1b and a digitized thicker cratonic area^59^. The transition to thicker or thinner areas is smoothed by a hyperbolic tangent with a half-width of 25 km.

### Rheology

Our description of the rheological model used to compute effective viscosity *η* in Eq. (1) consists of a viscoplastic rheology that combines the composite contributions of diffusion and dislocation creep with Drucker–Prager plasticity^91^. In addition, we include linear strain weakening of the internal angles of friction.

Diffusion and dislocation creep are formulated as^92^

5 $${\eta }_{{\rm{eff}}}^{{\rm{diff}}| {\rm{disl}}}=\frac{1}{2}{\left(\frac{1}{A}\right)}^{1/n}{\dot{\epsilon }}_{e}^{(1-n)/n}\exp \left(\frac{Q+PV}{nRT}\right),$$

where in the case of diffusion creep, *n* = 1, while for dislocation creep *n* > 1. The effective deviatoric strain rate is defined as ${\dot{\epsilon }}_{e}$ = $\sqrt{\frac{1}{2}{\dot{\epsilon }}_{ij}^{\prime}{\dot{\epsilon }}_{ij}^{\prime}}$. See Supplementary Table 1 for the definition and values of other symbols; the upper crust follows a wet quartzite rheological law^93^, the lower crust is described by wet anorthite^94^, while all mantle material behaves like dry olivine^95^.

Plastic yielding is implemented by locally rescaling the effective viscosity in such a way that the stress remains on the yield envelope^96^, with the effective plastic viscosity given by

6 $${\eta }_{{\rm{eff}}}^{{\rm{pl}}}=\frac{\frac{6C\cos \phi }{\sqrt{3}(3+\sin \phi )}+\frac{6P\sin \phi }{\sqrt{3}(3+\sin \phi )}}{2\dot{{\epsilon }_{e}}}.$$

The internal angle of friction *ϕ* is linearly weakened from 20° to 5° on the accumulated plastic strain *ϵ* interval [0.0, 0.5]^51,97,98^. Plastic strain is tracked on a compositional field as

7 $${\phi }_{{\rm{weakened}}}=\phi +(\phi -\phi \cdot {\phi }_{{\rm{wf}}})\left(\frac{\min (\max (\epsilon ,{\epsilon }_{\min }),{\epsilon }_{\max })-{\epsilon }_{\min }}{{\epsilon }_{\min }-{\epsilon }_{\max }}\right).$$

The resulting nonlinearities in the Stokes equations introduced by the viscoplastic rheology are iterated out using Picard iterations^99^.

### Boundary conditions

We use prescribed velocities representing far-field plate motions on the western and eastern boundaries to enforce rifting over time. As demonstrated by the evolving views on EARS development^9,74,100,101^, the timing of initiation and propagation of the eastern and western rift branches is complex. Nevertheless, there is general agreement that rifting was established in both branches by ~10 Ma^10,101,102^.

From geodetic and geological data inversion, the present-day Somalia–Nubia plate system relative extension direction is  ~E–W, as plotted in Fig. 1a along the eastern model domain boundary, and thus derived extension rates along the EARS reach up to 5.2 ± 0.9 mm yr^−1^, with earlier studies predicting up to 7.2 mm yr^−1^^3^. Good agreement is reached between this present-day motion and predictions from oceanic paleomagnetic data for the last 3.2 My^2,103^. A slowdown of Somalia–Nubia motion occurred around 11 Ma, but since then spreading velocities have been relatively stable, between 3.7 and 5.0 mm yr^−1^ with an azimuth of 80–120° for a point at 35.2°E, −18°N^104^. From Southwest Indian Ridge reconstructions, stable rift-normal extension has been found to occur since 5.2 Ma at 40^∘^E, 9^∘^N, concurrent with present-day motions^105^. Between 5.2 and 11 Ma, extension might have been slower, with velocities varying between 2.4 and 5.12 mm yr^−1^ over the last 11 My, and the extension direction between 105 and 125° from north.

Based on the above, we simplify our extensional boundary conditions to orthogonal, constant velocities^32,63^, applied such that the material is moving outward with a velocity of ${v}_{x}=\frac{1}{2}\cdot 5\,{\rm{mm}}\ {{\rm{yr}}}^{-1}$ on the right and left boundary (the tangential velocity components are left free). This outflow is compensated by inflow of an equal volume of material through the bottom boundary, and the front and back boundaries feature free slip. The top boundary is a true free surface to allow for the formation of topography in response to the internal stress state. Note that as we ignore the much smaller boundary-parallel component (~0.5 mm yr^−1^), in some places, this might reduce the obliquity of the extension direction, while increasing it in others. As oblique extension has been suggested to be more efficient^106^, this could change the localization of deformation and, in turn, the microplate rotation. Neglecting the small (≤20%) temporal changes in extension direction as suggested by plate reconstructions^104,105^ might similarly change the present-day velocity prediction.

### Post processing

To quantify our model results, we compute the rotation pole of the microplate between the eastern and western rift branches relative to the western (Nubian) plate. This is done by querying the finite-element solution for the velocity values at 3-km depth (w.r.t to the initial unperturbed surface) on a regularly spaced grid covering the entire domain and a more refined grid around the rift. For all points falling within the area spanned by the original mobile belt segments (up to a 100-km distance to these segments), we then invert for the Euler pole by first subtracting the rotation’s pivot point obtained through the least-squares method from all velocity point coordinates and then solving **v** = *A***Ω**, where **v** are the relative velocity vectors and **Ω** is the rotation vector^1^. An estimate of the confidence of the location of the pivot point and thus of the rigidity of the microplate is obtained from the root-mean-square residual distance (RMSD) between the point and the lines normal to the velocities. In comparing our model poles with those found from data inversion, it is important to remember that the inversion of GNSS data, earthquake slip vectors, and geologic indicators assumes that the plates are rigid blocks, with only some studies^3^ accounting for elastic strain accumulating on plate-boundary faults. Our models, however, do not include elastic deformation, but do allow for internal deformation of the plates.

On the same regular grid, we also compute the maximum horizontal compressive stress ${\sigma }_{{\rm{Hmax}}}$ and the tectonic regime (normal faulting NF, normal faulting with a strike–slip component NS, strike–slip faulting SS, thrust faulting with a strike–slip component TS, or thrust faulting TF) from the plunges of the compressive stress eigenvectors^107^. Note that this is the convention used in the World Stress Map Project^55^. We further track the displacement along the rift segments with passive particles advected with the flow. Initially, these particles are distributed in pairs along the rift on opposing sides and at 200-km distance of the  mobile belt axis. After 10 My of model time, we decompose the pairwise relative motion vector into a rift-parallel and -normal component.

## Supplementary information

Supplementary Information

## Data Availability

The input files to reproduce the data that support the findings in this study have been deposited in the GitHub repository https://github.com/anne-glerum/paper-Victoria-microplate-rotation.

## Code availability

The modeling software ASPECT can be obtained from GitHub at https://github.com/geodynamics/aspect; in this study, a modification of a 2.1.0-predeveloper version was used (commit 31a88da) that can be obtained from https://github.com/anne-glerum/aspect/tree/paper-Victoria-microplate-rotation. The repository https://github.com/anne-glerum/paper-Victoria-microplate-rotation contains the input files to reproduce the model results, as well as information on the versions of the libraries underlying the ASPECT installation.
