# Supplementary material for: Victoria continental microplate dynamics controlled by the lithospheric strength distribution of the East African Rift
Source: Nat Commun. 2020 Jun 8;11:2881. doi: 10.1038/s41467-020-16176-x (PMC7280495; doi:10.1038/s41467-020-16176-x)
Supplement: Supplementary file 1 — Supplementary Information [file 41467_2020_16176_MOESM1_ESM.pdf]

VICTORIA CONTINENTAL MICROPLATE DYNAMICS CONTROLLED BY THE LITHOSPHERIC STRENGTH DISTRIBUTION OF THE EAST AFRICAN RIFT

ANNE GLERUM[1]*

SASCHA BRUNE[1,2]

D. SARAH STAMPS[3]

MANFRED STRECKER[2]

[1] *Helmholtz Centre Potsdam - GFZ German Research Centre for Geosciences, Telegrafenberg, 14473 Potsdam,* Germany
[2] *University of Potsdam, Karl-Liebknecht-Str. 24-25, 14476 Potsdam-Golm,* Germany
[3] *Virginia Tech, 4044 Derring Hall, 926 West Campus Drive Blacksburg, VA 24061,* United States

E-mail addresses: *acglerum@gfz-potsdam.de.

1. Supplementary Information

The below Supplementary Table 1 provides the values of the material properties of the compositional fields that represent the upper crust, lower crust, lithospheric mantle and sublithospheric mantle in the models presented in this study. The upper crust follows a wet quartzite rheological law[1], the lower crust is described by wet anorthite[2], while all mantle material behaves like dry olivine[3]. The internal angle of friction ϕ is linearly weakened[4,5,6] from 20° to 5° on the accumulated plastic strain ϵ interval [0.0,0.5].

Supplementary Table 1 Material property definitions and reference values for each model composition.

Property	Upper crust	Lower crust	Lithospheric mantle	Sublithospheric mantle	Unit
Reference temperature T_0	293	293	293	293	K
Reference density ρ_0	2700	2850	3280	3300	$\mathrm{kg\,m^{-3}}$
Thermal expansivity α	$2.7 \cdot 10^{-5}$	$2.7 \cdot 10^{-5}$	$3 \cdot 10^{-5}$	$3 \cdot 10^{-5}$	$\mathrm{K^{-1}}$
Thermal diffusivity κ	$7.72 \cdot 10^{-7}$	$7.31 \cdot 10^{-7}$	$8.38 \cdot 10^{-7}$	$8.33 \cdot 10^{-7}$	$\mathrm{m^2\,s^{-1}}$
Heat capacity C_p	1200	1200	1200	1200	$\mathrm{J\,kg^{-1}\,K^{-1}}$
Radioactive heating H	$1.5 \cdot 10^{-6}$	$0.2 \cdot 10^{-6}$	0	0	$\mathrm{W\,m^{-3}}$
Prefactor A_{diff}	$5.97 \cdot 10^{-19}$	$2.99 \cdot 10^{-25}$	$2.25 \cdot 10^{-9}$	$2.25 \cdot 10^{-9}$	$\mathrm{Pa^{-1}\,s^{-1}}$
Activation volume V_{diff}	0	$38 \cdot 10^{-6}$	$6 \cdot 10^{-6}$	$6 \cdot 10^{-6}$	$\mathrm{m^3\,mol^{-1}}$
Activation energy Q_{diff}	$223 \cdot 10^3$	$159 \cdot 10^3$	$375 \cdot 10^3$	$375 \cdot 10^3$	$\mathrm{J\,mol^{-1}}$
Prefactor A_{disl}	$8.57 \cdot 10^{-28}$	$7.13 \cdot 10^{-18}$	$6.52 \cdot 10^{-16}$	$6.52 \cdot 10^{-16}$	$\mathrm{Pa^{-n}\,s^{-1}}$
Activation volume V_{disl}	0	$38 \cdot 10^{-6}$	$18 \cdot 10^{-6}$	$18 \cdot 10^{-6}$	$\mathrm{m^3\,mol^{-1}}$
Activation energy Q_{disl}	$223 \cdot 10^3$	$345 \cdot 10^3$	$530 \cdot 10^3$	$530 \cdot 10^3$	$\mathrm{J\,mol^{-1}}$
Stress exponent n	4.0	3.0	3.5	3.5	-
Cohesion C	$20 \cdot 10^6$	$20 \cdot 10^6$	$20 \cdot 10^6$	$20 \cdot 10^6$	Pa
Internal friction angle ϕ	20	20	20	20	°
Strain weakening interval [ϵ_{min},ϵ_{max}]	[0.0,0.5]	[0.0,0.5]	[0.0,0.5]	[0.0,0.5]	-
ϕ weakening factor ϕ_{wf}	0.25	0.25	0.25	0.25	-
Unperturbed thickness d	20	15	85	180	km

Abbreviations: diff = diffusion creep, disl = dislocation creep

References

[1] Rutter, E. H. & Brodie, K. H. Experimental grain size-sensitive flow of hot-pressed Brazilian quartz aggregates. *Journal of Structural Geology* **26**, 2011–2023 (2004).

[2] Rybacki, E., Gottschalk, M., Wirth, R. & Dresen, G. Influence of water fugacity and activation volume on the flow properties of fine-grained anorthite aggregates. *Journal of Geophysical Research: Solid Earth* **111** (2006). URL `http:https://dx.doi.org/10.1029/2005JB003663`.

[3] Hirth, G. & Kohlstedt, D. Rheology of the upper mantle and the mantle wedge: a view from the experimentalists. In Eiler, J. (ed.) *Inside the Subduction Factory*, vol. 183 of *Geophysical Monograph* (American Geophysical Union, 2003).

[4] Huismans, R. S. & Beaumont, C. Symmetric and asymmetric lithospheric extension: Relative effects of frictional-plastic and viscous strain softening. *Journal of Geophysical Research* **108** (2003).

[5] Naliboff, J. & Buiter, S. J. H. Rift activation and migration during multiphase extension. *Earth and Planetary Science Letters* **421**, 58–67 (2015).

[6] Le Pourhiet, L., May, D. A., Huille, L., Watremez, L. & Leroy, S. A genetic link between transform and hyper-extended margins. *Earth and Planetary Science Letters* **465**, 184–192 (2017).
